# Induction of Apoptosis in Human Breast Cancer Cells via Caspase Pathway by Vernodalin Isolated from *Centratherum anthelminticum* (L.) Seeds

**DOI:** 10.1371/journal.pone.0056643

**Published:** 2013-02-20

**Authors:** Chung Yeng Looi, Aditya Arya, Foo Kit Cheah, Bushra Muharram, Kok Hoong Leong, Khalit Mohamad, Won Fen Wong, Nitika Rai, Mohd Rais Mustafa

**Affiliations:** 1 Department of Pharmacology, Faculty of Medicine, University of Malaya, Kuala Lumpur, Malaysia; 2 Department of Pharmacy, Faculty of Medicine, University of Malaya, Kuala Lumpur, Malaysia; 3 Department of Medical Microbiology, Faculty of Medicine, University of Malaya, Kuala Lumpur, Malaysia; 4 Amritum Bio-Botanica Herbs Research Laboratory Pvt. Ltd., Jogli, India; University of California Irvine, United States of America

## Abstract

**Background:**

*Centratherum anthelminticum* (L.) seeds (CA) is a well known medicinal herb in Indian sub-continent. We recently reported anti-oxidant property of chloroform fraction of *Centratherum anthelminticum* (L.) seeds (CACF) by inhibiting tumor necrosis factor-α (TNF-α)-induced growth of human breast cancer cells. However, the active compounds in CACF have not been investigated previously.

**Methodology/Principal Findings:**

In this study, we showed that CACF inhibited growth of MCF-7 human breast cancer cells. CACF induced apoptosis in MCF-7 cells as marked by cell size shrinkage, deformed cytoskeletal structure and DNA fragmentation. To identify the cytotoxic compound, CACF was subjected to bioassay-guided fractionation which yielded 6 fractions. CACF fraction A and B (CACF-A, -B) demonstrated highest activity among all the fractions. Further HPLC isolation, NMR and LC-MS analysis of CACF-A led to identification of vernodalin as the cytotoxic agent in CACF-A, and -B. 12,13-dihydroxyoleic acid, another major compound in CACF-C fraction was isolated for the first time from *Centratherum anthelminticum* (L.) seeds but showed no cytotoxic effect against MCF-7 cells. Vernodalin inhibited cell growth of human breast cancer cells MCF-7 and MDA-MB-231 by induction of cell cycle arrest and apoptosis. Increased of reactive oxygen species (ROS) production, coupled with downregulation of anti-apoptotic molecules (Bcl-2, Bcl-xL) led to reduction of mitochondrial membrane potential (MMP) and release of cytochrome c in both human breast cancer cells treated with vernodalin. Release of cytochrome c from mitochondria to cytosol triggered activation of caspase cascade, PARP cleavage, DNA damage and eventually cell death.

**Conclusions/Significance:**

To the best of our knowledge, this is the first comprehensive study on cytotoxic and apoptotic mechanism of vernodalin isolated from the *Centratherum anthelminticum* (L.) seeds in human breast cancer cells. Overall, our data suggest a potential therapeutic value of vernodalin to be further developed as new anti-cancer drug.

## Introduction

Breast cancer is one of the most common malignancies in women. Global breast cancer incidence has increased at an annual rate of 3.1% over the last three decades to more than 1.6 million cases in year 2010 [1]. In Malaysia, breast cancer is the most common cancer among females. There were 3,242 female breast cancer cases diagnosed in 2007, accounted for 18.1% of all cancer cases reported and 32.1% of all female cases (National Cancer Registry Report 2007). Different subtypes of breast cancers arise from different gene mutations occurring in luminal or basal progenitor cell population, causing difficulty in breast cancer diagnosis and treatment [2]. Being both genetically and histopathologically heterogeneous, the mechanisms underlying breast cancer development remains uncertain [3]. Owing to this, conventional chemotherapy, surgery or radiation shows very limited effects. On the other hand, specific natural or synthetic chemical compounds have been widely applied for cancer chemoprevention to inhibit or revert carcinogenesis and to suppress the malignancy of cancer [4].

Medicinal plants have been used for centuries to treat a variety of diseases and maintain health before the advent of modern medicine [5], [6]. The accumulation and developing knowledge of the medicinal properties of plants by personal experimentation, local custom, anecdote, and folk tradition leads to the formation of numerous traditional medical systems and therapies, including traditional Chinese medicine (TCM), Ayurvedic medicine, indigenous medicine, naturopathy and aromatherapy [7], [8], [9]. In modern medicine, plants have been a source for new anti-cancer drugs. For example, vinblastine was traditionally obtained from *Catharanthus roseus*, taxol was isolated from the bark of the Pacific yew tree *Taxus brevifolia*, camptothecin was isolated from the bark and stem of *Camptotheca acuminata* [10], [11], [12]. The advancement of technology such as gas chromatography-mass spectrometry (GC-MS) and liquid chromatography-mass spectrometry (LC-MS) have speed up the process of drug screening and discovery [13]. LC-MS is a highly sensitivity and selectivity method used in drug development at many different stages including profiling of secondary metabolites in plants, impurities detection, metabolic stability or degradant analysis [14], [15].

**Figure 1:**
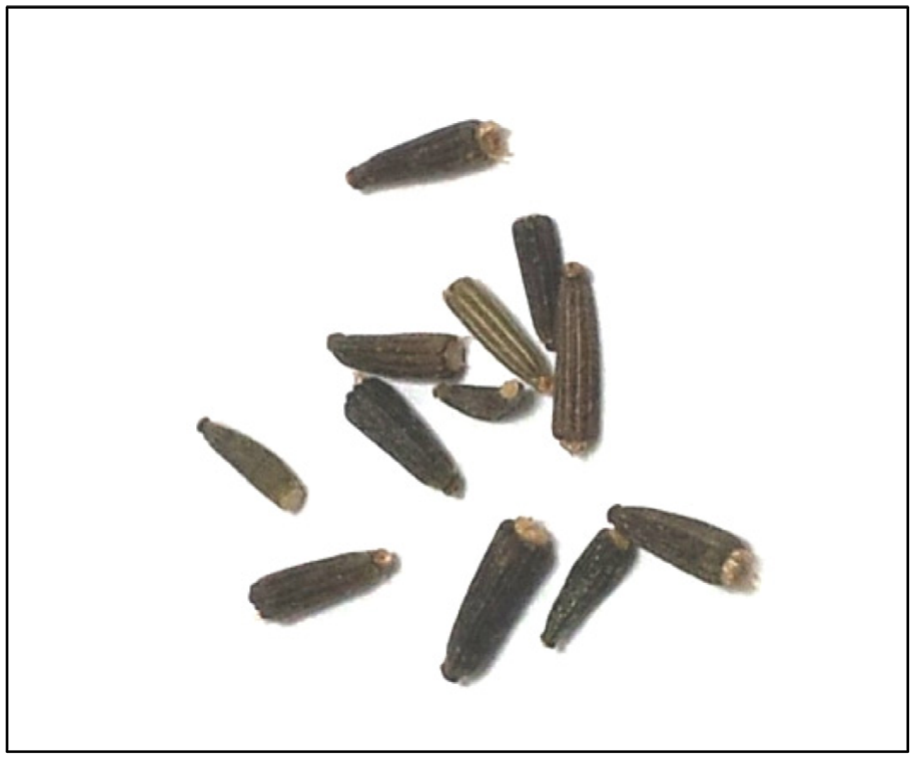
Photo of *Centratherum anthelminticum (L.)* seeds.

*Centratherum anthelminticum* (L.) Kuntze, commonly known as kalajiri, somraj, black cumin or bitter cumin, is a robust leafy plant belongs to Asteraceae family of the flowering plants (Figure 1). Scientific synonyms for this plant include *Vernonia anthelmintica* and *Conyza anthelmintica*. This plant can be found in India, Himalaya mountain, Khasi mountain, Sri Lanka, Afghanistan, and is widely used as a traditional herb against fever, cough and diarrhea in the region. Recent experimental analyses have proven that extracts from seeds of *C. anthelminticum* possess various pharmacological properties. The methanolic extract from the *C. anthelminticum* seeds demonstrates antiviral properties [16] whereas acetone and ethyl acetate extracts demonstrate antifilarial activity against *Setaria cervi* [17]. Besides, petroleum ether and alcohol extracts show analgesic, antipyretic and anti-inflammatory effect in rat model [18], [19]. Different extracts from *C. anthelminticum* seeds also show antimicrobial and antifungal properties when screened on various pathogens *in vitro* [20]. A recent report also suggests *C. anthelminticum* seeds phenols inhibit liposomal peroxidation and protect oxidative damage to genomic DNA of Bacillus, therefore can function as an anti-oxidant agent [21].

In 2004, Lambertini *et al.* reported the *in vitro* anti-proliferative effect of extracts from *C. anthelminticum* on human breast cancer cells [22]. We recently reported that the chloroform, but not hexane or methanol fractions from *C. anthelminticum* (L.) seeds (CACF) exhibited anti-oxidant property by inhibiting tumor necrosis factor-α (TNF-α)-induced human cancer cell growth by interrupting the activation of nuclear factor-kappa B (NF-κB) [23]. However, the active compounds in CACF were not examined in the previous reports. In this study, we showed that CACF inhibited MCF-7 breast cancer cell growth. Administration of CACF caused morphological changes, disrupted cytoskeletal structures and DNA fragmentation. Bioassay-guided fractionation led us to the identification of vernodalin as the cytotoxic agent in CACF. To the best of our knowledge, this is the first report on the cytotoxic and the apoptotic mechanism of vernodalin isolated from *C. anthelminticum* seeds in human breast cancer cells.

## Materials and Methods

### Plant Material

The seeds of *C. anthelminticum* were purchased from the medicinal plant cultivation zone of Amritum Bio-Botanica Herbs Research Laboratory Pvt. Ltd, Betul Madhya Pradesh India. The seeds were identified by the quality control department of the company itself. Voucher specimen (CA-9) was deposited at the Pharmacology Department of University Malaya, Malaysia.

### Cell Culture

The human breast cancer cell line, MCF-7 was purchased from Cell Lines Service (300273; Eppelheim, Germany) and MDA-MB-231 cell line was obtained from American Type Culture Collection (HTB-26; ATCC, Manassas, VA). Human mammary epithelial cells were purchased from ScienCell (7610; Carlsbad, CA) and maintained in mammary epithelial cell medium (ScienCell). MCF-7 and MDA-MB-231 cells were grown in Dulbecco’s Modified Eagle Medium (DMEM, Life Technologies, Inc, Rockville, MD) supplemented with 10% heat-inactivated fetal bovine serum (Sigma-Aldrich, St. Louis, MO), 2 mM glutamine, 1% penicillin and streptomycin. Cells were cultured in tissue culture flasks (Corning, USA) and were kept in CO_2_ incubator at 37°C in a humidified atmosphere with 5% CO_2_. For experimental purposes, cells in exponential growth phase (approximately 70–80% confluency) were used.

### Extraction and Isolation

The powdered seeds of *C. anthelminticum* (100 g) were extracted successively with hexane (3×250 ml) (Merck, Darmstadt, Germany), chloroform (CHCl_3_) (3×250 ml) (Merck, Darmstadt, Germany) and methanol (MeOH) (3×250 ml) (Merck, Darmstadt, Germany), in a Soxhlet apparatus for 24 hours. The resultant extracts were filtered using Whatman No. 1 filter paper (Whatman, England) and dried under vacuum to yield 20.1, 7.7, 11.6 g, respectively of the extracts. Then the dried fractions were kept at −20°C until further use. In our previous paper, the chloroform extract (CACF) showed highest activity on MTT assay, therefore CACF was chosen for this study [23].

### Bioassay Guided Isolation

The chloroform extract was fractionated using reversed phase C_18_ (Merck, Germany) flash column chromatography. The column was preconditioned with water, then the extract was added to the column and eluted using a step gradient of water and methanol as follows: MeOH:H_2_O (1∶1, 3×100 ml); MeOH:H_2_O (6∶4, 3×100 ml); MeOH:H_2_O (7∶3, 3×100 ml); MeOH:H_2_O (8∶2, 3×100 ml); MeOH:H_2_O (9∶1, 3×100 ml) and MeOH (10∶0, 5×100 ml). The fractions were dried using a rotary evaporator. Similar fractions were pooled according to their liquid chromatography mass spectrometry (LC-MS) profile using Shimadzu UFLC-IT-TOFMS, into six fractions (CACF-A, CACF-B, CACF-C,……CACF-F). Each fraction was tested for their cytotoxic activity using MTT assay on MCF-7 cell line.

The cytotoxic active fractions CACF-A, CACF-B and CACF-C were then further purified using either preparative high performance liquid chromatography (HPLC) (Gilson GX-281/322 system) using a Waters Novapak C_18_ column (25×100 mm, 6 µm) or by recrystallisation. The major active compound of the fractions CACF-A and CACF-B were obtained by preparative HPLC. The fractions were eluted at a flow rate of 12 ml per min over 75 min. The gradient started at 10% solvent (A) (acetonitrile with 0.1% formic acid) and 90% solvent B (water with 0.1% formic acid) for 5 min. The gradient then changed from 10% to 60% (A) over 5 minutes, followed by 60–100% (A) for 50 minutes and finally an isocratic elution of 100% (A) from 60 to 75 min. The fractions yielded 10 mg of compound **(1)** (colorless oil) eluted at about 30 min. Fraction CACF-C was purified by recrystallization from ethyl acetate (EtOAC) (Merck, Darmstadt, Germany)/diethyl ether (Merck, Darmstadt, Germany) to give a white powder of compound **(2)** (183.5 mg).

### Identification of Compounds

The ^1^H and ^13^C Nuclear Magnetic Resonance (NMR) spectroscopy were carried out on a JOEL NMR 400 and 100 MHz with TMS as internal standard. HR-ESI-MS was measured on a LCMS-IT-TOF mass spectrometer (Shimadzu IT-TOF). MS was recorded on Shimadzu GC-MS model QP2010 Plus spectrophotometer.

### Spectral Data

Vernodalin **(1)** was obtained as colorless oil. The ^1^H and ^13^C NMR spectral data obtained for CDCl_3_ solution of the compound were in agreement to those previously identified as vernodalin [24]. The positive ion HR-ESI-MS spectrum showed a molecular ion peak at *m/z* 361 [M+H]^+^ consistent to molecular formula C_19_H_21_O_7_. ^1^H NMR (CDCl_3_, 400 MHz): δ 6.74 (1H, s, H-15α), 6.29 (1H, s, H-4′α), 6.20 (1H, d, H-13α), 5.96 (2H, s, H-4′β, H-15 β), 5.71(1H, dd, *J* = 11, 17.4, H-1), 5.64 (1H, d, *J* = 3.2,H-13 β), 5.32 (2H, dd, *J* = 6.84,11, H-2), 5.13 (1H, td, *J = *4.56, 10.52,H-8), 4.48 (1H, d, *J* = 12.36, H-14 α), 4.35 (1H, s, H-3′), 4.28 (1H, dd, *J* = 1.36, 12.36, H-14β), 4.06 (1H, t, *J* = 11, H-6), 3.03 (2H, m, H-5, H-7), 2.24 (1H, dd, *J* = 4.6, 14.2, H-9), 2.11 (1H, br s, OH), 1.71 (1H, dd, *J* = 10.52, 14.2, H-9). ^13^C NMR (CDCl_3_, 100 MHz): δ 168.4 (C-12), 165.1 (C-1′), 163.2 (C-3), 139.6 (C-2′), 138.8 (C-1), 136.0 (C-15), 135.6 (C-11), 129.9 (C-4), 127.2 (C-4′), 121.7 (C-13), 117.2 (C-2). 78.1 (C-6), 70.6 (C-14), 68.8 (C-8), 62 (C-3′), 50.5 (C-7), 46.7 (C-5), 41.1 (C-10), 39.0 (C-9).

12,13-dihydroxyoleic acid **(2),** was obtained as white powder. The LCMS-IT-TOF spectra showed molecular ion peaks, [M-H]^-^ at *m/z* 313 consistent to molecular formula C_18_H_34_O_4_. ^1^H NMR (CDCl_3_, 400 MHz): δ 5.6 (1H, m, H-10), 5.4 (1H, m, H-9), 3.49 (2H, q, *J* = 2.4, 4.8, H-11, H-12), 2.36 (4H, m, H-8, H-11), 2.07 (2H, q, *J = *6.4, 14.0, H-2), 1.65 (2H, m, H-3), 1.51-1.32 (16H, m, (CH_2_)_8_), 0.91 (3H, t, *J* = 6.8, H-18). ^13^C NMR (CDCl_3_, 100 MHz): δ 178 (C-1), 133.6 (C-9), 124.7 (C-10), 73.9 (C-12), 73.8 (C-13), δ 14.0–33.7 (CH_2_)_12_, 14.0 (C-18).

### MTT Cell Viability Assay

1×10^4^ cells per well were seeded into 96-well plate overnight. Cells were treated with various concentrations of compound or extract (dissolved in dimethyl sulfoxide, DMSO) for 24 hours. As negative control, cells were treated with vehicle (DMSO) only. Next, cells were incubated with 50 µl of 4,5-dimethylthiazol-2-yl-2,5-diphenyltetrazolium bromide (MTT) (2 mg/ml) at 37°C for 2 hours. After dissolving the formazan crystals in DMSO, plates were read in Chameleon™ multitechnology microplate reader (Hidex, Turku, Finland) at 570 nm against 620 nm. This experiment was performed in triplicates and repeated for 3 times. Mean values ± SD for each concentration was determined. Calculation of cell viability was described previously with slight modification [25]. Cell viability (in percentages, %) was showed as ratio of absorbance (A_570 nm_) in treated cells relative to absorbance in control cells (DMSO) (A_570 nm_). The IC_50_ was defined as the concentration of sample needed to reduce 50% of absorbance relative to the vehicle (DMSO)-treated control.
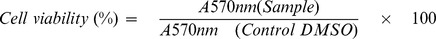



### Real Time Cell Growth Assay

Cell proliferation was measured using xCELLigence Real-Time Cellular Analysis (RTCA) system (Roche, Germany), which allows us to monitor the cell viability and cell growth continuously at multiple time point. Briefly, background measurements were taken after adding 50 µl of the culture medium to the wells. Next, cells were seeded at density 1×10^4^ on a specialized 16-well plate with electrodes for 18 hours to allow cells grow to the log phase. Cells were treated with 100 µl of CACF or vernodalin in various concentrations (µg/ml) dissolved in cell culture media and continuously monitored for up to 72 hours. Cell sensor impedance was expressed as an arbitrary unit called the Cell Index. Cell index were recorded every 5–10 minutes by RTCA analyzer. To eliminate variation between wells, cell index values were normalized to the value at the beginning of treatment time-point.

### Real Time Cell Invasion Assay

The kinetics of cell invasion was assayed using the xCELLigence Real-Time Cell Analyzer (RTCA DP; Roche). CIM-plates (Roche) were pre-coated with 30 µl of matrigel (BD Biosciences) diluted 1∶10 in DMEM for 1 h at 37°C. The upper chambers contained pre-warmed serum-free DMEM, whereas the lower chambers contained either DMEM with 10% FCS or DMEM medium only (negative control). Indicated concentrations of vernodalin were added into the medium of upper and lower chambers. 1×10^4^ MDA-MB-231 cells were seeded into each well of the upper chambers. Cells were allowed to settle for 30 min at room temperature before being placed back to the RTCA DP in a humidified incubator at 37°C with 5% CO_2_. Readings were taken every 10 min for 16 h and plotted curves represent the averages from two independent wells per measurement.

### Apoptosis Assay

For *in vitro* fluorescent staining, 1×10^4^ cells per well were seeded in 96 well-plate overnight. Cells were then treated with CACF at various concentrations for 12 hours. Live cells were stained with FITC-annexin V (BD Biosciences, San Jose, CA) for 15 minutes before fixed with 4% paraformaldehyde. Cells were washed 3 times with PBS and the fluorescent images were acquired using Cellomics ArrayScan high content screening (HCS) reader (Thermo Scientific, Pittsburgh, PA). Compartmental analysis bioapplication module was used to quantify the fluorescence intensity of FITC-annexin V.

For apoptosis assay by flow cytometry, cells were seeded at 1×10^5^ per ml on 25 cm^2^ flask overnight before treated with vernodalin at various concentrations for 24 hours. Determination of apoptotic cells by fluorescent staining was done as described previously [25]. Briefly, cells were incubated with FITC-annexin V and propidium iodide (PI) (BD Biosciences) in binding buffer for 15 minutes in dark. Stained cells were immediately subjected to flow cytometry analyses using FACS Canto II flow cytometer (BD Biosciences).

### Cytoskeletal Rearrangement Analysis

1×10^4^ MCF-7 cells per well seeded overnight in 96-well plate were exposed to DMSO (negative control) or CACF at various concentrations for 12 hours. Cells were fixed, washed with wash buffer before probed using phalloidin conjugated with DyLight™ 554 and Hoechst 33258 according to the manufacturer’s instruction. Cells were visualized and images were acquired using Cellomics ArrayScan HCS reader (Thermo Scientific). Morphology bioapplication module was used to quantify the fluorescence intensity of phalloidin.

### Cell Cycle Analysis

1×10^5^ cells per ml seeded overnight in 25 cm^2^ flask were treated with vernodalin for 24 hours. Cells were then fixed with 70% ethanol overnight. Cells were washed twice with PBS and stained with CycleTEST™ PLUS DNA Reagent Kit (BD Biosciences) according to manufacturer’s instructions. Cell cycle distribution of nuclear DNA was determined by flow cytometry (BD Biosciences) by analyzing at least 20,000 cells per sample. The percentage of cells in G1, S and G2 phases were analyzed by Diva software (BD Biosciences).

### Reactive Oxygen Species (ROS) Analysis

1×10^4^ cells per well were seeded onto 96-well plate. Cells were treated with vernodalin or DMSO (negative control) at indicated concentrations for 12 hours. Dihydroethidium (DHE) dye contained in Cellomics ROS kit was added into live culture for 30 minutes. Cells were fixed and washed with wash buffer as described by the manufacturer’s instruction. Stained cells were visualized and acquired using Cellomics ArrayScan HCS reader (Thermo Scientific). Target activation bioapplication module was used to quantify the fluorescence intensities of DHE dye in the nucleus.

### Nuclear Morphology, Membrane Permeability, Mitochondrial Membrane Potential Δψm (MMP) and Cytochrome C Release Analysis

Cellomics Multiparameter Cytotoxicity 3 Kit (Thermo Scientific) was used. Cells were plated at 1×10^4^ cells per well on 96-well plate overnight. DMSO (solvent) or vernodalin was added at various concentrations and further incubated for 24 hours. MMP dye (Excitation 552/Emission 576) and the cell permeability dye (Excitation 491/Emission 509) were added to live cells and incubated for 1 hour. Cells were fixed with 4% formaldehyde for 15 minutes. Fixed cells were permeabilized with 0.1% Triton X-100 in phosphate buffer saline (PBS). Samples were blocked with 3% bovine serum albumin and incubated with cytochrome c primary mouse antibody for 1 hour. Samples were washed three times with wash buffer I (1×PBS) before addition of goat anti-mouse secondary antibodies conjugated with DyLight™ 649. Cells were rinsed three times with wash buffer II (1×PBS with 1% Tween-20). Nucleus was stained with Hoechst 33258. Stained cells were visualized and images were captured using Cellomics ArrayScan HCS reader (Thermo Scientific). Cell health profiling bioapplication module was used to quantify the fluorescence intensities of each dye.

### Western Blot Analysis

SDS-PAGE and Western blot analyses were done as described with slight modifications [26]. Briefly, 24 hours post treatment, cells were lysed in RIPA buffer (1% NP-40, 0.5% sodium deoxycholate, 0.1% SDS) supplemented with freshly added 10 mM β-glycerophosphate, 1 mM sodium orthovanadate, 10 mM NaF, 1 mM phenylmethylsulfonyl fluoride and Protease Inhibitor Cocktail (Santa Cruz, CA) and loaded onto 10% polyacrylamide gel. Proteins were then transferred to microporous polyvinylidene difluoride (PVDF) membrane (Milipore). Membranes were incubated in 5% BSA (Sigma) blocking buffer for 1 h at room temperature. Incubations with primary antibody were carried out overnight at 4°C. Immunoblotting was performed with the following antibodies: rabbit anti-cleaved caspase-3, anti-cleaved caspase-7, anti-cleaved caspase-9, anti-cleaved PARP, anti-Bcl-2, anti-Bcl-xL (1∶200) (Cell Signaling Technology, Danvers, MA), and mouse anti-β-actin (1∶500) (Sigma) antibodies. Membranes were washed 3 times (10 min each) in Tween buffer before incubating with HRP-conjugated goat anti-mouse or rabbit secondary antibodies. To remove excess antibodies, membranes were washed 4 times before HRP activities were detected using ECL Plus Chemiluminescence Reagent (Amersham, Chalfont, UK) according to the protocol supplied with the kit.

### Bioluminescent Assays for Caspase-3/7,-8 and -9 Activities

A time-dependent study of caspase-3/7, -8 and -9 activities was performed in triplicates using assay kits Caspase-Glo® 3/7, 8 and 9 (Promega, Madison, WI) on a white 96-well microplate. A total of 1×10^4^ cells was seeded per well and incubated with 100 µl of vernodalin (final concentration 6.25 µg/ml) for 1, 3, 6, 12, 18, 24 and 30 hours. Caspase activities were investigated according to the manufacture protocol. Briefly, 100 µl caspase-Glo reagent was added and incubated at room temperature for 30 minutes. Presences of active caspases from apoptotic cells cleaved the aminoluciferin-labeled synthetic tetrapeptide thus release substrate for the luciferase enzyme. The caspase activities were measured using a Tecan Infinite®200 Pro (Tecan, Männedorf, Switzerland) microplate reader.

### Statistical Analysis

Experimental values were expressed as the means ± standard deviation (SD) of the number of experiments indicated in the legends. Analysis of variance (ANOVA) was performed using GraphPad Prism 5 software. Statistical significance was defined when *P*<0.05.

## Results

### CACF Inhibits Survival of Human Breast Cancer MCF-7 Cells

**Figure 2:**
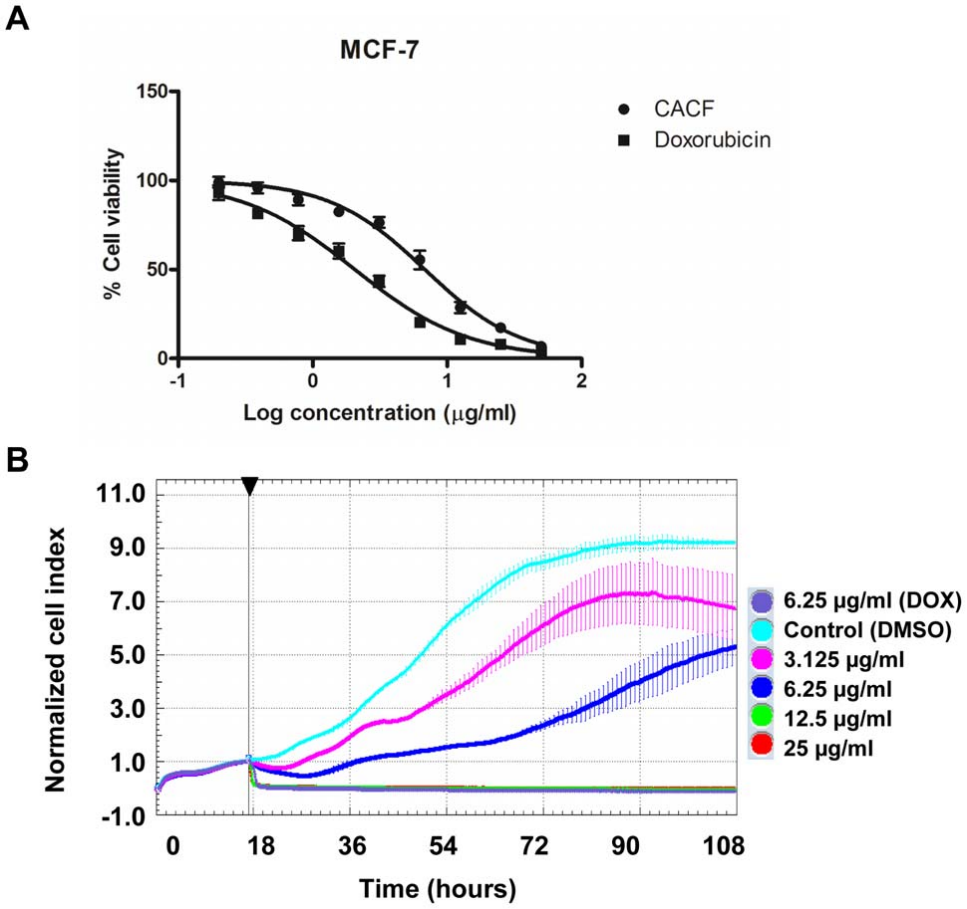
CACF inhibits MCF-7 cells proliferation in a time- and dose-dependent manner. (A) MCF-7 cells were treated with control DMSO, various concentrations (0.195, 0.39, 0.78, 1.56, 3.125, 6.25, 12.5, 25, 50 µg/ml) of CACF or anti-cancer drug doxorubicin for 24 hours. Cell viability was determined by MTT assays. (B) Real-time cell proliferation was measured using xCELLigence Real-Time Cellular Analysis (RTCA) system. MCF-7 cells were treated with DMSO (control), indicated concentration of CACF or doxorubicin (DOX) and normalized cell index for 3 consecutive treatment days was shown. Data were mean ± SD. Arrow showing time-point of CACF administration.

We first determined the cytotoxic effect of CACF on cell survival using a well-characterized human breast cancer cell line, MCF-7. MTT assay was used to determine cell viability. The survival of MCF-7 decreased significantly in a concentration dependent manner with IC_50_ value at 6.8±1.2 µg/ml (Figure 2A). No significant cell inhibitory effect was observed in DMSO (solvent)-treated samples. As a positive control, we treated MCF-7 cells with doxorubicin, a cancer chemotherapy drug, which showed IC_50_ value at 2.0±0.8 µg/ml. To verify MTT results, we repeated the experiments using Alamar blue staining for cell viability. We found comparable results between MTT and Alamar blue staining assays (Figure S1A).

MTT assays are end point assays which only detect cell viability at certain time-point. Next, we observed the subtle changes or the pattern of cell growth after CACF-treatment for 3 consecutive days using real-time cell proliferation assay (RTCA). In control wells (vehicle, DMSO only), we observed an exponential increased of cell growth as reflected by increased in normalized cell index (nCI) values. Whereas MCF-7 treated with doxorubicin at concentration 6.25 µg/ml resulted in cell growth inhibition (Figure 2B). A dose-dependent attenuation of cell proliferation was observed in CACF-treated MCF-7 (Figure 2B). As shown in Figure 2B, we observed a sudden decrease in nCI values about 1–2 hours after treated with 25 or 12.5 µg/ml of CACF, indicating acute toxicity at high dosages. Together, our results showed that CACF inhibited cell growth of MCF-7 breast cancer cells in dose- and time-dependent manners.

### Morphological Assessment of CACF-treated MCF-7 Cells

**Figure 3:**
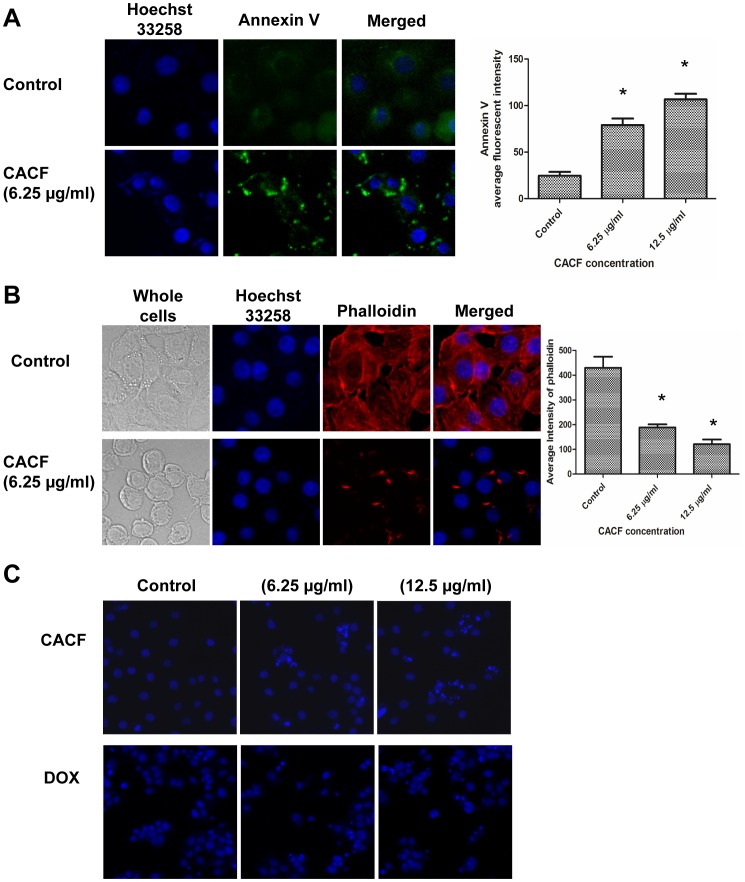
Morphological assessment of CACF-treated MCF-7 cells. (A) Representative figures of MCF-7 cells were treated with CACF for 12 hours. Cells were stained with apoptosis marker annexin V (green) and nucleus marker Hoechst 33258 (blue). Histogram shows mean fluorescence intensities of annexin V in MCF-7 cells treated with various concentration of CACF. Data were mean ± SD, *P<0.05. (B) Representative figures of cytoskeletal F-actin formation in control or CACF-treated MCF-7 cells. Cells were fixed, stained with DY544-phalloidin (red) and Hoechst 33258 (blue) after treated with 6.25 µg/ml CACF or solvent DMSO for 12 hours. Histogram shows mean fluorescence intensities of phalloidin in MCF-7 cells treated with various concentration of CACF. Data were mean ± SD, *P<0.05. (C). Representative figures of MCF-7 cells treated with DMSO (control), 6.25 or 12.5 µg/ml of CACF for 24 hours. Cells were also treated with a standard drug doxorubixin (DOX) as positive control of apoptosis induction. Cells were stained with Hoechst 33258 dye (blue). All images were visualized and captured using Cellomic HCS array scan reader (objective 20 ×).

Next, we examined if CACF treatment resulted in cell death through apoptotic pathway. We treated MCF-7 cells with control (DMSO solvent) or CACF for 12 hours before staining live cells with apoptosis marker annexin V conjugated to FITC. Exposure of 6.25 and 12.5 µg/ml of CACF led to higher annexin V staining compared to control, suggesting apoptotic activities (Figure 3A).

Under light miscroscope, we observed that MCF-7 cells exposed to CACF resulted in reduction of cell size and cell-cell contact areas (Figure 3B). To further investigate this, we examined cytoskeletal F-actin structure by staining the cells with phalloidin conjugated to DyLight™ 554, which detect polymerized actin (F-actin). Control cells demonstrated well-organized actin filament bundles or stress fibers in the cytoplasm (Figure 3B). On the contrary, CACF treatment (6.25 and 12.5 µg/ml) on MCF-7 cells caused a drastic reduction in phalloidin stain (Figure 3B) and loss of stress fibers in the cytoplasm. Furthermore, F-actin was no longer distributed evenly at the cell periphery, but appeared as punctuate stain at the plasma membrane. This result suggests that CACF treatment led to the disruption of cytoskeletal structure in MCF-7 cells.

Because apoptotic activity is usually associated with DNA cleavage, we examined the effect of CACF on nuclear morphology of MCF-7 cells using Hoechst 33258. After CACF treatment for 24 hours, a population of condensed and fragmented nuclei was observed (Figure 3C). The number of cells with fragmented nuclei increased with higher dosages of CACF administrated while no detectable DNA damage was detected in control cells. Together, these data indicated that CACF treatment triggered apoptotic pathway as evidenced by higher annexin V staining, cell shrinkage, disrupted cytoskeleton and DNA damage in MCF-7 cells.

### Cytotoxic Activities of CACF Fractions

**Figure 4:**
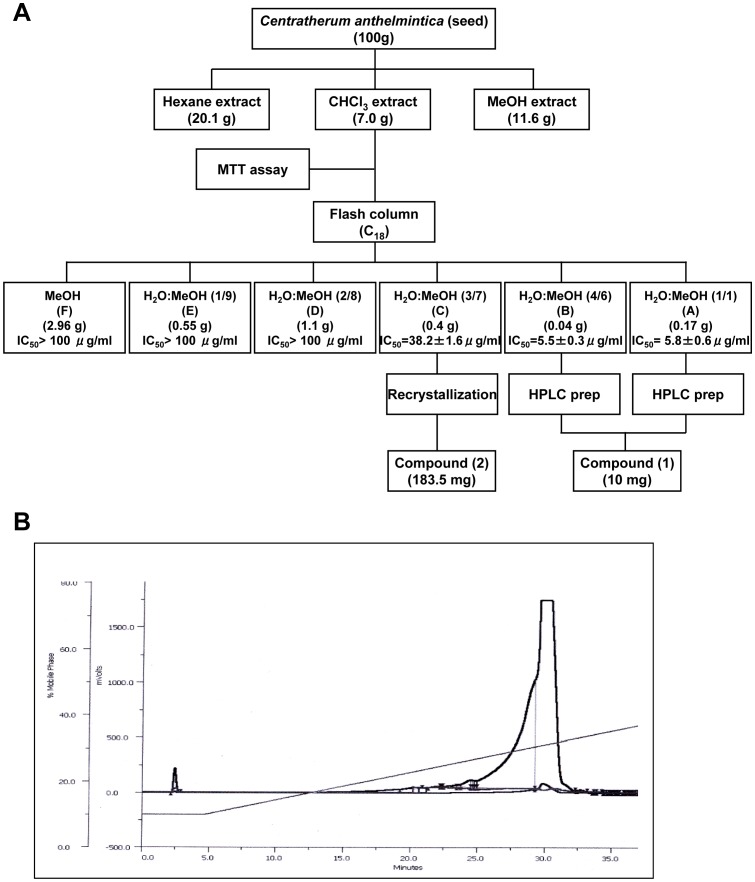
Isolation of active compound from CACF. A. Flow chart of bioassay guided isolation of *Centratherum anthelminticum*. B. HPLC chromatogram of the fraction of CACF-A of the chloroform extract of *C. anthelminticum*.

To identify the active compound in CACF extracts which possesses cytotoxicity activity against human breast cancer cells, we performed HPLC analysis. The cytotoxic extract CACF was fractionated into six fractions using preparative HPLC (Figure 4A and 4B). Among these fractions, CACF-A and CACF-B showed highest activity on MCF-7 cells with IC_50_ values of 5.8±0.6 µg/ml and 5.5±0.3 µg/ml, respectively. However, CACF-C showed moderate activity on MCF-7 cells with IC_50_ value of 38.2±1.6 µg/ml while the other fractions (CACF- D to F) exhibited low cytotoxic activity (IC_50_>100 µg/ml). Compounds in fractions CACF-A, -B and -C were further isolated as described in Materials and Methods.

**Figure 5:**
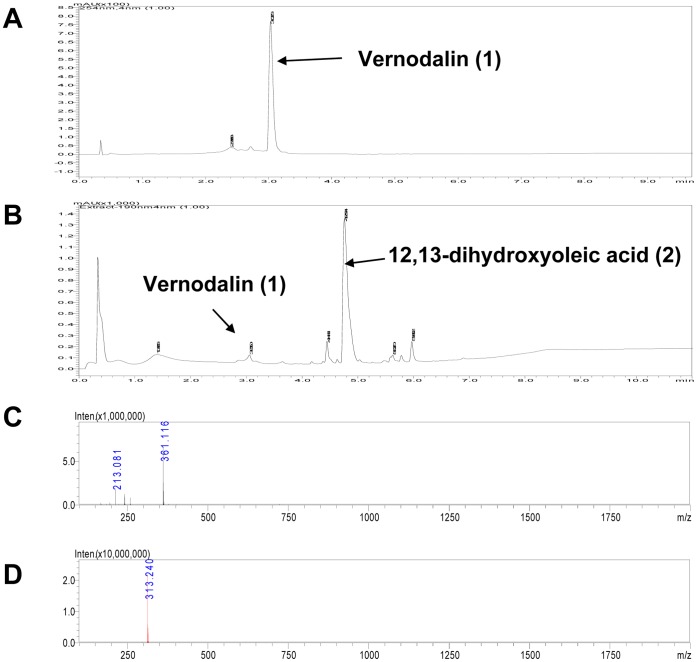
Mass spectra of CACF isolated fractions. (A, B) LC-MS chromatograms of fraction CACF-A and CACF-C. (A) Single peak detected in the fraction CACF-A was identified as vernodalin **(1)**. (B) Major peak detected in fraction CACF-C was identified as 12,13-dihydroxyoleic acid **(2)** while vernodalin **(1)** constituted a small part in the fraction. (C) HR-ESI-MS spectrum (positive mode) of vernodalin **(1)**. (D) HR-ESI-MS spectrum (negative mode) of 12,13-dihydroxyoleic acid **(2)**.

**Figure 6:**
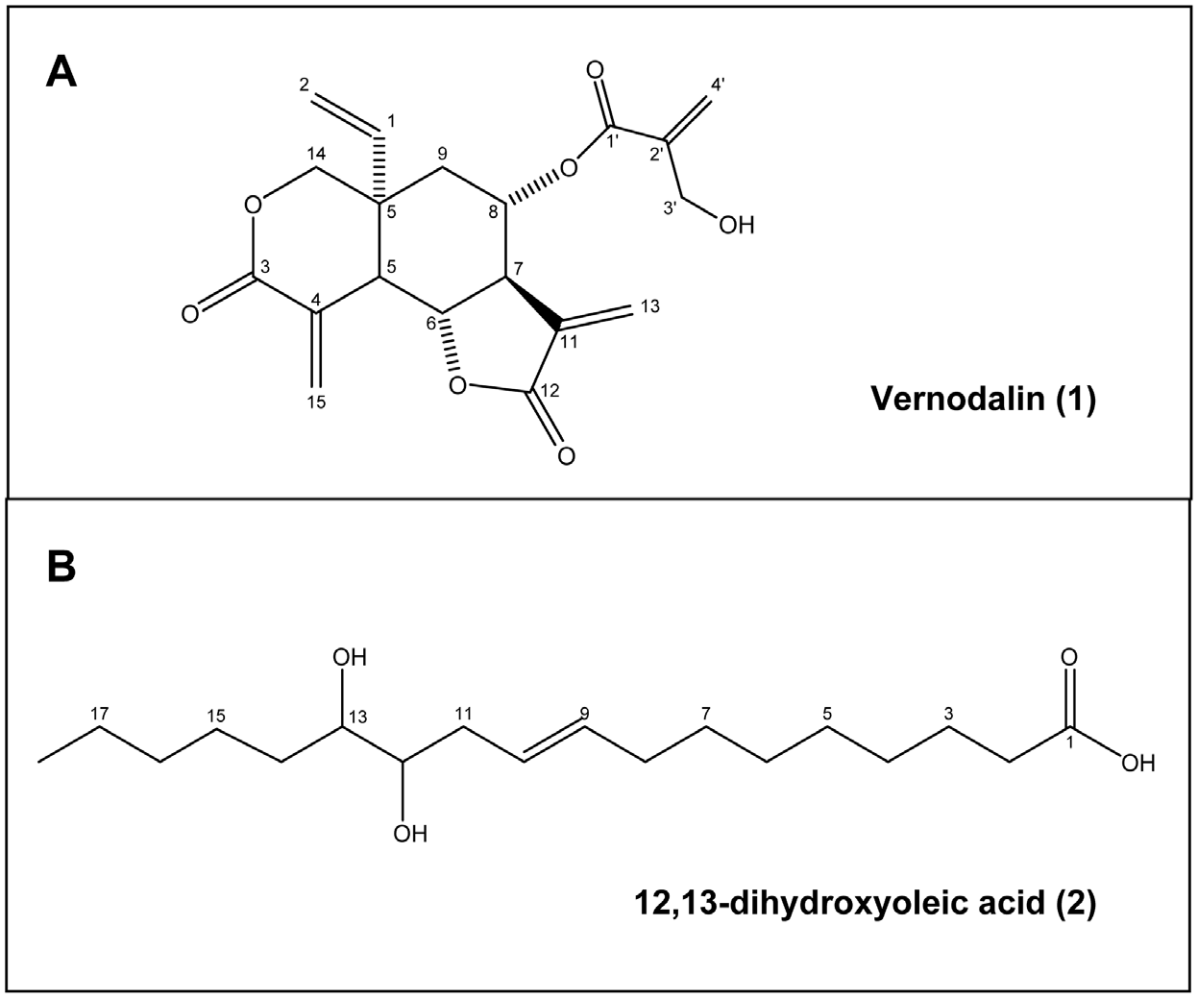
Chemical structure of vernodalin (1) and 12,13-dihydroxyoleic acid (2).

Major compounds in the CACF-A, -B and -C fractions were subjected to LC-MS analysis. LC-MS analysis showed that vernodalin **(1)** (10 mg), eluted at 3.0 min, was the major compound of CACF-A and CACF-B fractions (Figure 5A and 5C). In addition, 12,13-dihydroxyoleic acid **(2)**, eluted at 4.75 min, (183.5 mg) was largely detected in CACF-C fraction while vernodalin was minor (1 mg) (Figure 5B and 5D). Chemical structures of vernodalin **(1)** and dihydroxyoleic acid **(2)** were depicted in Figure 6A and 6B.

### Vernodalin Inhibits Cell Growth of MCF-7 and MDA-MB-231 Cells

**Figure 7:**
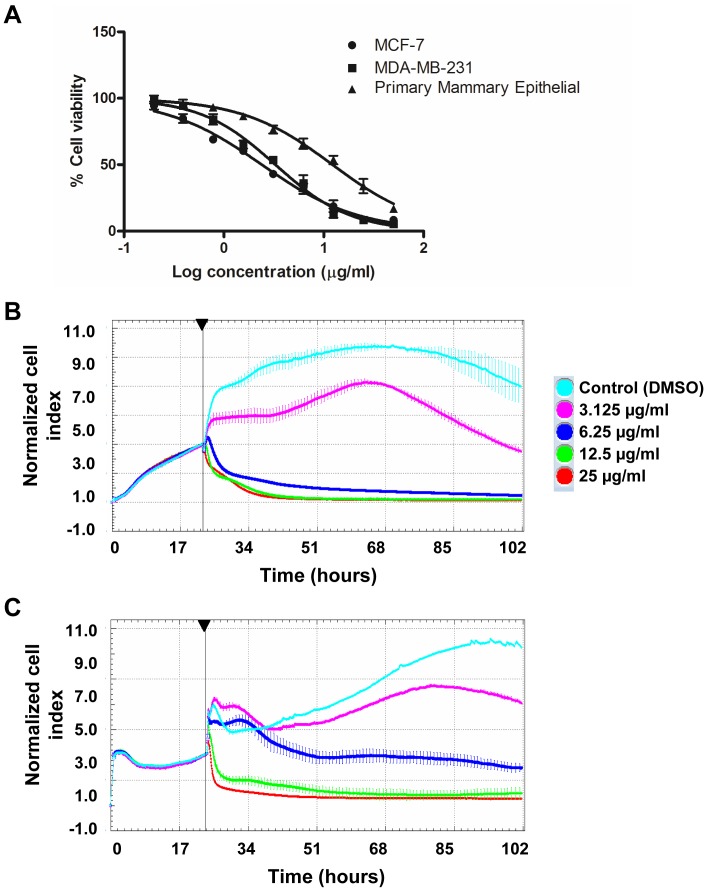
Vernodalin inhibits proliferation of MCF-7 and MDA-MB-231 human breast cancer cell lines. (A) MCF-7, MDA-MB-231 and primary mammary epithelial cells were treated with vehicle (DMSO) or various concentrations (0.195, 0.39, 0.78, 1.56, 3.125, 6.25, 12.5, 25, 50 µg/ml) of vernodalin for 24 hours. Cell viability was determined by MTT assays. (B) Real-time cell growth was determined using RTCA analyzer. MCF-7 and MDA-MB-231 cells were treated with DMSO (control) or indicated concentrations of vernodalin. Normalized cell index for 3 consecutive treatment days was shown for each sample. Data were mean ± SD. Arrow showing time-point of vernodalin administration.

To examine the *in vitro* anti-cancer efficacy of vernodalin, we included a highly invasive and metastatic variant of human breast cancer cell-line, MDA-MB-231, apart from MCF-7 (non-metastatic). Both MCF-7 and MDA-MB-231 cells were exposed to various concentrations of vernodalin for 24 hours. Cell viability was determined by MTT assays. The IC_50_ values for vernodalin treated MCF-7 and MDA-MB-231 were 2.5±0.3 µg/ml and 3.4±0.6 µg/ml, respectively (Figure 7A). On the other hand, the IC_50_ of normal mammary epithelial cells was 12.7±0.5 µg/ml, relatively more resistant to cell killing by vernodalin. We further verified the results using Alamar blue proliferation assay (Figure S1B). Meanwhile, 12,13-dihydroxyoleic acid, another compound isolated showed no cytotoxicity effect on both breast cancer cell lines, IC_50_>100 µg/ml (data not shown).

Next, we monitored real-time vernodalin-mediated cell growth inhibition for 3 consecutive days by RTCA. As shown in Figure 7B and 7C, a dose-dependent cell-growth inhibition was observed in vernodalin-treated MCF-7 and MDA-MB-231 cells. Reduced nCI was observed at concentration 3.125 µg/ml, whereas no significant increment in nCI at 6.25 µg/ml or higher after vernodalin treatment compared to control (Figure 7B and 7C). These results showed that vernodalin inhibited cell growth of breast cancer cells, MCF-7 and MDA-MB-231 in a dose- and time-dependent manner.

### Vernodalin Inhibits Invasive Potential of Metastatic Breast Cancer Cells

**Figure 8:**
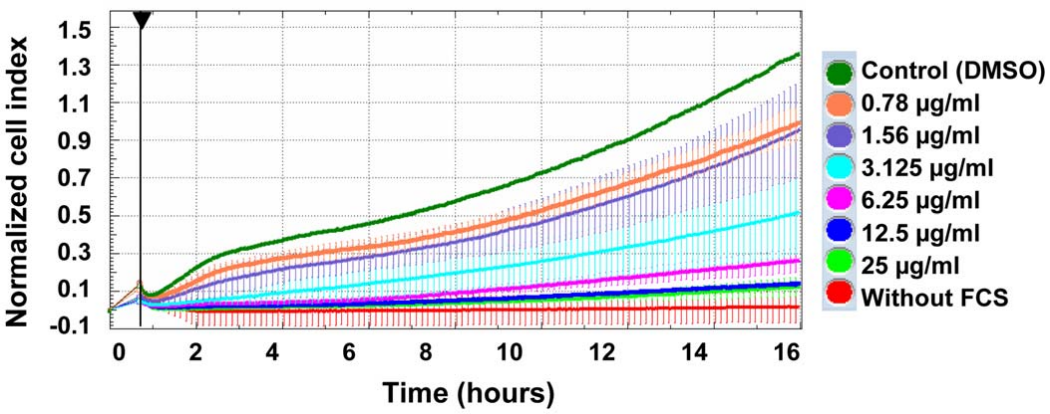
Vernodalin inhibits invasion of MDA-MB-231 human breast cancer cell line. Real-time cell invasive assay. MDA-MB-231 cells were seeded into upper chamber of CIM plates coated with matrigel. Lower chamber were filled with medium with FCS or medium only. Cells were treated with DMSO (control) or indicated concentrations of vernodalin and continuously monitored for 16 hours. Increased cell migration to lower chamber resulted in higher normalized cell index. Data were mean ± SD from two independent experiments.

Next, we examined whether vernodalin has an influence on the invasive potential of metastatic breast cancer cells MDA-MB-231 by using modified Boyden chamber (CIM plates, Roche) with matrigel as a substrate. The presence of chemoattractant FCS strongly induced non-treated MDA-MB-231 cell migration to the lower chamber. In contrast, no signal or cell invasion was detected in lower chamber without addition of FCS. On the other hand, dose-dependent inhibition of cell invasion was observed upon vernodalin treatment (Figure 8). Of note, we previously showed that CACF treatment resulted in reduced phalloidin staining (Figure 3B), implying that vernodalin-mediated cell invasion inhibition was probably mediated through disruption of actin polymerization.

### Vernodalin causes Apoptosis and Cell Cycle Arrest

**Figure 9:**
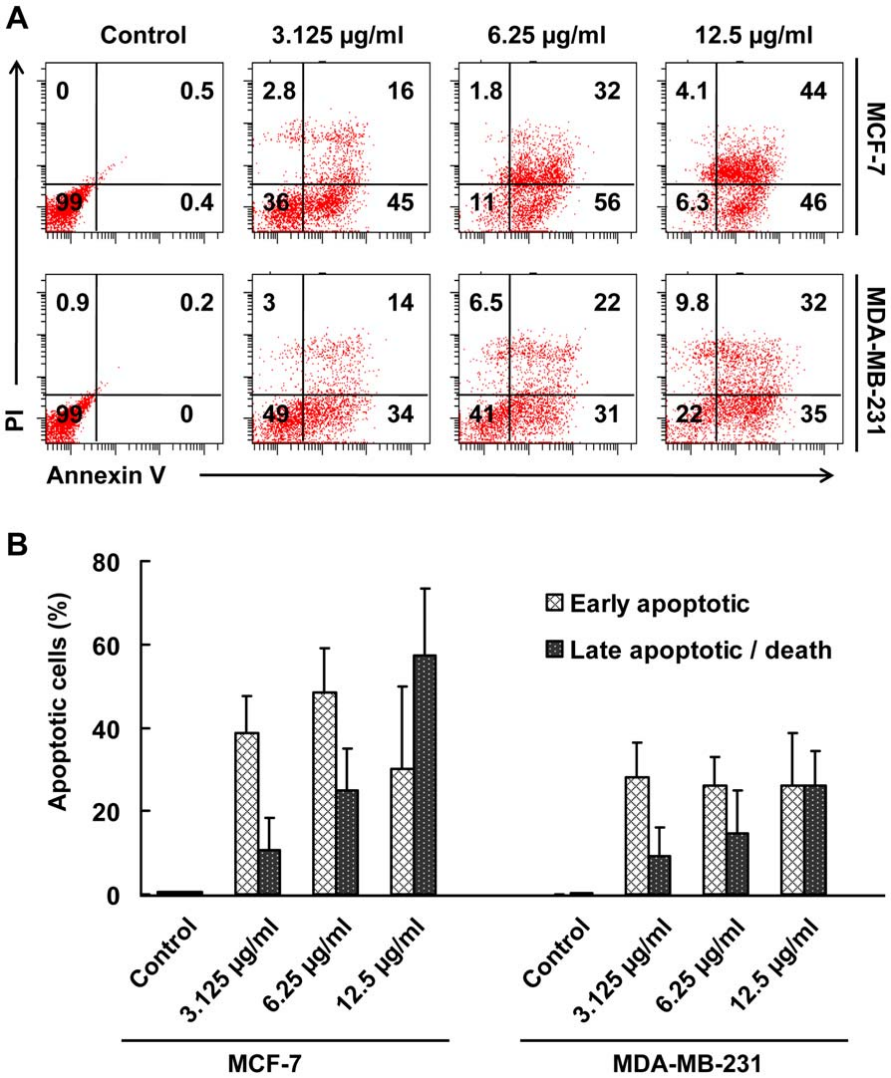
Vernodalin induces apoptosis in human breast cancer cells. (A) Flow cytometry analysis of MCF-7 and MDA-MB-231 cells treated with 3.125, 6.25 and 12.5 µg/ml verdonalin for 24 hours. Representative figures showing population of viable (annexin V- PI-), early apoptotic (annexin V+ PI-), late apoptotic (annexin V+ PI+) and necrotic (annexin V- PI+) cells. (B) Bar chart showing increased proportion of early and late apoptotic cells after vernodalin administration. Data were mean ± SD of two independent experiments. (*P<0.05).

To examine whether cells undergo apoptosis, untreated or vernodalin-treated MCF-7 and MDA-MB-231 breast cancer cells were stained with annexin V and PI. Flow cytometry analysis of stained cells can distinguish cells into four groups, namely viable (annexin V- PI-), early apoptosis (annexin V+ PI-), late apoptosis (annexin V+ PI+) and necrotic (annexin V- PI+) cells. As shown in Figure 9A and 9B, vernodalin exposure at different concentrations (3.125, 6.25 and 12.5 µg/ml) resulted in higher population of early apoptotic population (30.0±19.7% to 48±10.8% in MCF-7 cells; 26.1±8.5% to 28.3±6.8% in MDA-MB-231 cells) compared to control (<1%). There were dose-dependent increments of late apoptotic population (10.5±7.7%, 25.1±9.8%, 57.4±16.0% in MCF-7 cells; and 9.1±6.8%, 14.7±10.2%, 25.9±8.5% in MDA-MB-231 cells) when treated with 3.125, 6.25 or 12.5 µg/ml of vernodalin. Less than 10% of population showed necrotic signs when treated with high dosage (12.5 µg/ml) of vernodalin.

**Figure 10:**
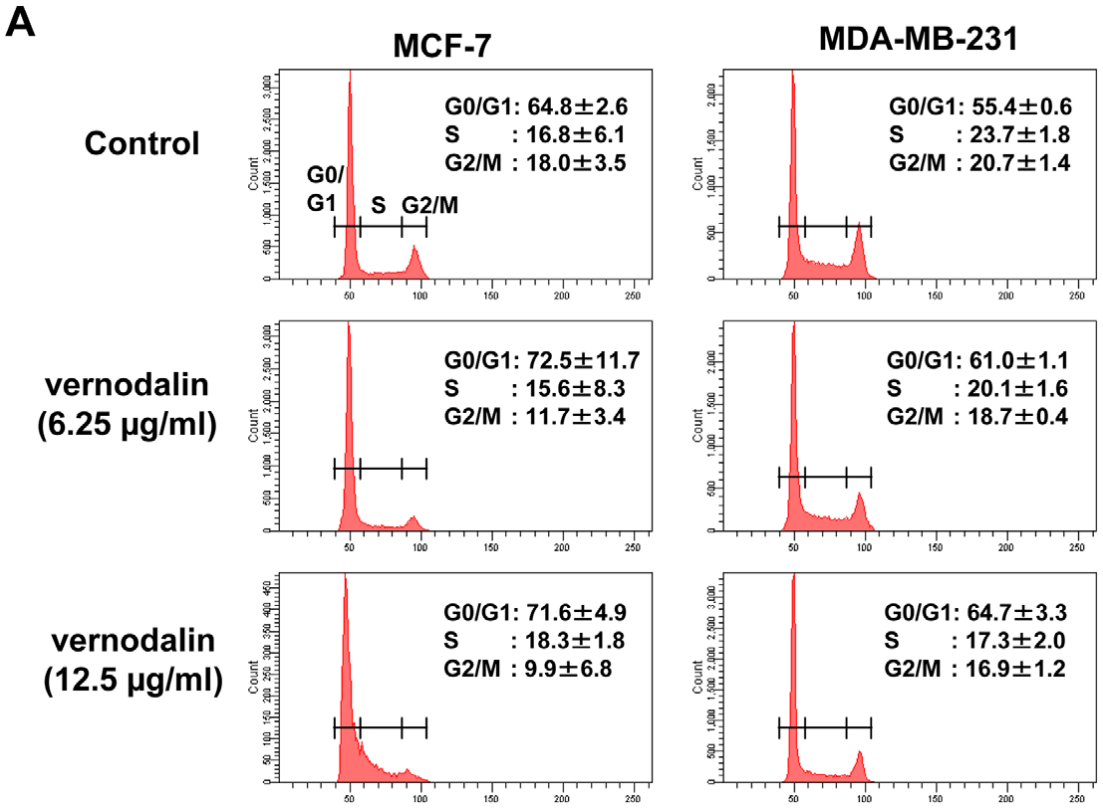
Vernodalin induces cell cycle arrest at G0/G1 stage. MCF-7 and MDA-MB-231 cells were treated with indicated dosages of verdonalin for 24 hours. Cells were ethanol-permeabilized and stained with propidium iodide before subjected to flow cytometry analysis. Representative figures of cell cyle distribution (G0/G1, S, and G2/M) showing accumulation of vernodalin-treated cells in G0–G1 stage. Data were mean ± SD of two independent experiments.

Next we examined the cell cycle distribution by staining vernodalin treated breast cancer cells with propidium iodide and analyzed the percentages of G0/G1, S, G2/M cell population using flow cytometry. MCF-7 cells treated with 6.125 or 12.5 µg/ml vernodalin showed higher G0/G1 population (72.5±11.7% and 71.6±4.9%, respectively) compared with 64.8±2.6% in the control (Figure 10). MDA-MB-231 cells treated with 6.125 or 12.5 µg/ml vernodalin also showed higher G0/G1 population (61.0±1.1% and 64.7±3.3%) compared to 55.4±0.6% in control cells. In addition, vernodalin treatment caused a concomitant decrease in the proportion of cells in G2/M phase of the cell cycle from control (18.0±3.5%) to treated MCF-7 cells (9.9±6.8% or 11.7±3.4%), and from control (20.7±1.4%) to treated MDA-MB-231 cells (16.9±1.2% or 18.7±0.4%). Therefore, our data suggest that vernodalin induced cell cycle arrest at the G0/G1 phase (Figure 10).

### Vernodalin Induces ROS Generation

**Figure 11:**
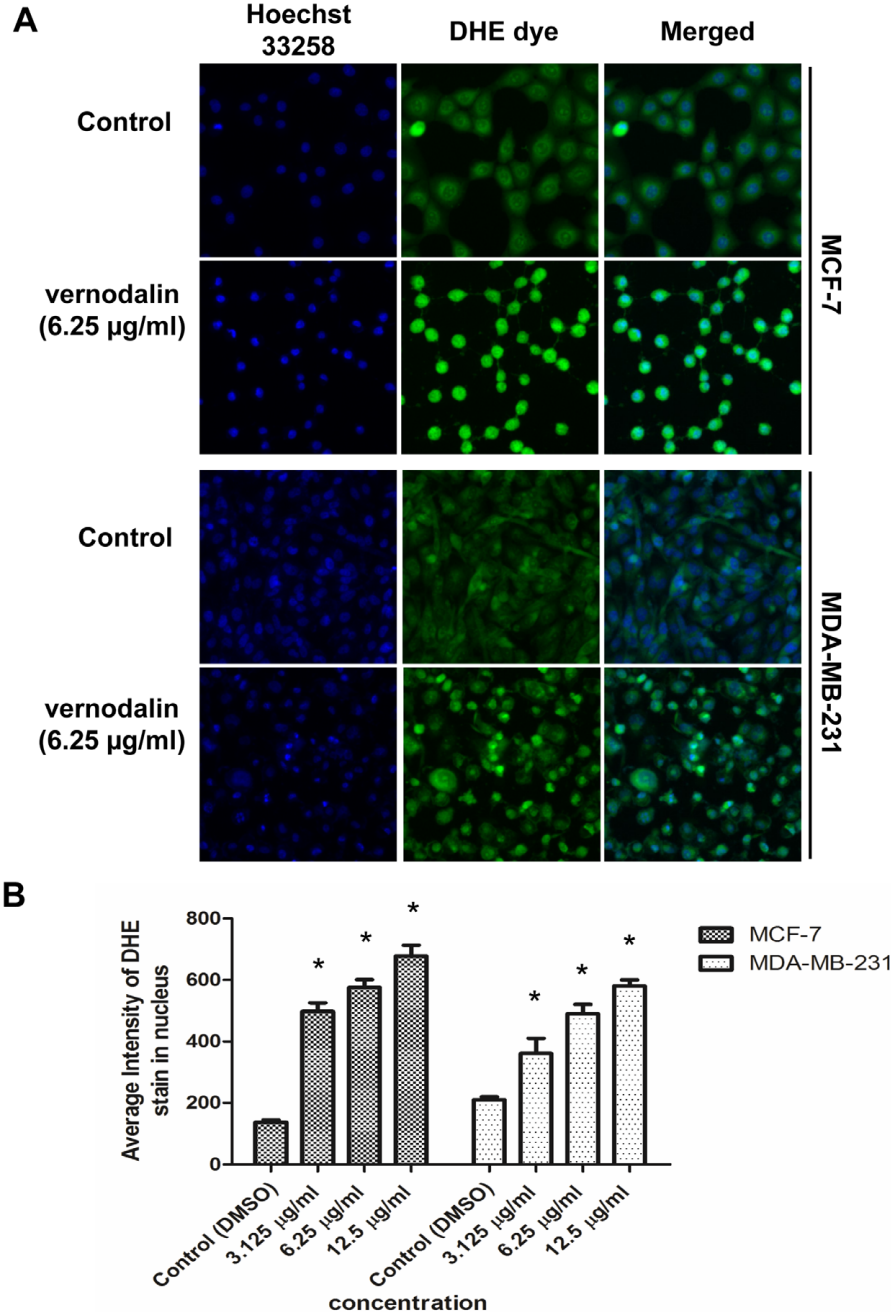
Vernodalin mediates ROS production. (A) MCF-7 or MDA-MB-231 cells were treated with DMSO (control) or indicated concentration of vernodalin for 12 hours. Live cells were stained with DHE dye (green) before cells were fixed and stained with Hoechst 33258 (blue). Images were acquired using Cellomic HCS array scan reader (objective 20 ×). Representative figures (control or 6.25 µg/ml vernodalin-treated) were shown. (B) Bar chart showing average fluorescence intensities of DHE dye in the nucleus. Data were mean ± SD of fluorescence intensity readings representative of three independent experiments. (*P<0.05).

ROS is produced especially when cells undergo chemical or environmental stress and could be one of the causative factors leading to cell cycle arrest or apoptosis. Next, we examined the ROS level in control or vernodalin-treated breast cancer cells by staining with DHE dye. ROS convert non-fluorescent DHE to fluorescent ethidium, which then intercalates into DNA. Hoechst 33258, a DNA binding dye is used to identify the nuclei of individual cells then the DHE fluorescence is quantified using the Cellomic HCS machine to evaluate the oxidative stress level. As shown in Figure 11A and 11B, ROS production was at the basal level in control DMSO-treated MCF-7 or MDA-MB-231 cells. In contrast, treatment with vernodalin (12 hours) resulted in dose-dependent increased of ROS production as shown by increased DHE staining in the nucleus (Figure 11A and 11B).

### Effect of Vernodalin on Nuclear Morphology, Membrane Permeabilization, MMP (Δψm) and Cytochrome c Release

**Figure 12:**
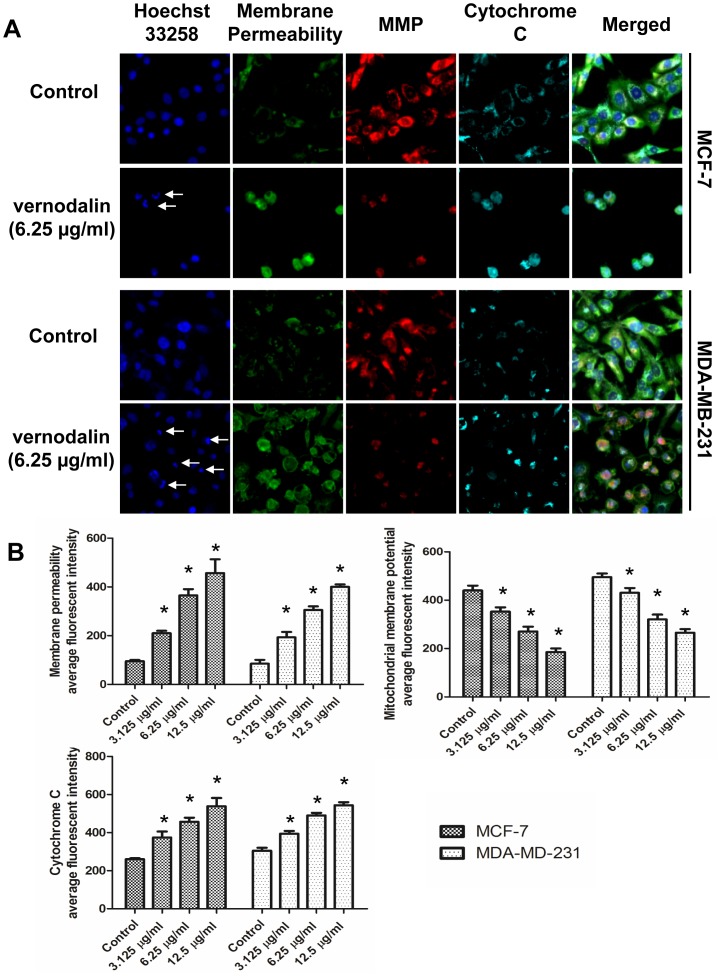
Effect of vernodalin on nuclear morphology, membrane permeabilization, MMP (Δψm) and cytochrome c release. MCF-7 or MDA-MB-231 cells were plated in 96-well plates and treated with either vehicle (DMSO) or indicated dosages of vernodalin for 24 hours. Cells were fixed and stained according to the manual. Images were acquired using Cellomic HCS array scan reader (objective 20 ×). (A) Representative figures showing changes in DNA content (blue), cell permeability (green), MMP (red) and cytochrome c (cyan). Arrows showed condensed or fragmented DNA. (B) Bar chart showing dose-dependent increased in cell permeability, reduced MMP and increased cytochrome c release in vernodalin-treated samples. Data were mean ± SD of fluorescence intensity readings of three independent experiments. (*P<0.05).

Since high ROS production could lead to plasma membrane, DNA, mitochondrial damage, we further examined the nuclear morphology, membrane permeability, mitochondrial membrane potential (MMP, Δψm) and cytochrome c release and localization. As shown in Figure 12A and 12B, 24 hours of exposure to vernodalin revealed a concentration-dependent increment of membrane permeability, attenuation of MMP and increased cytochrome c in the cytosol compared to control. In some vernodalin-treated cells, cytochrome c was localized in the nucleus and we could observe nuclear condensation and fragmentation in these cells (Figure 12A).Whereas in control samples, nucleus remained rounded and uniform in size. Moreover, plasma membrane was intact as shown by the weak staining of permeability dye (green, Figure 12A). Cytochrome c (cyan) was distributed homogenously in the cytosol, which colocalized with MMP dye (red), indicating that cytochrome c was not released from the mitochondria in control cells (Figure 12A).

### Effect of Vernodalin Treatment on Caspase-3/7, -8, -9

**Figure 13:**
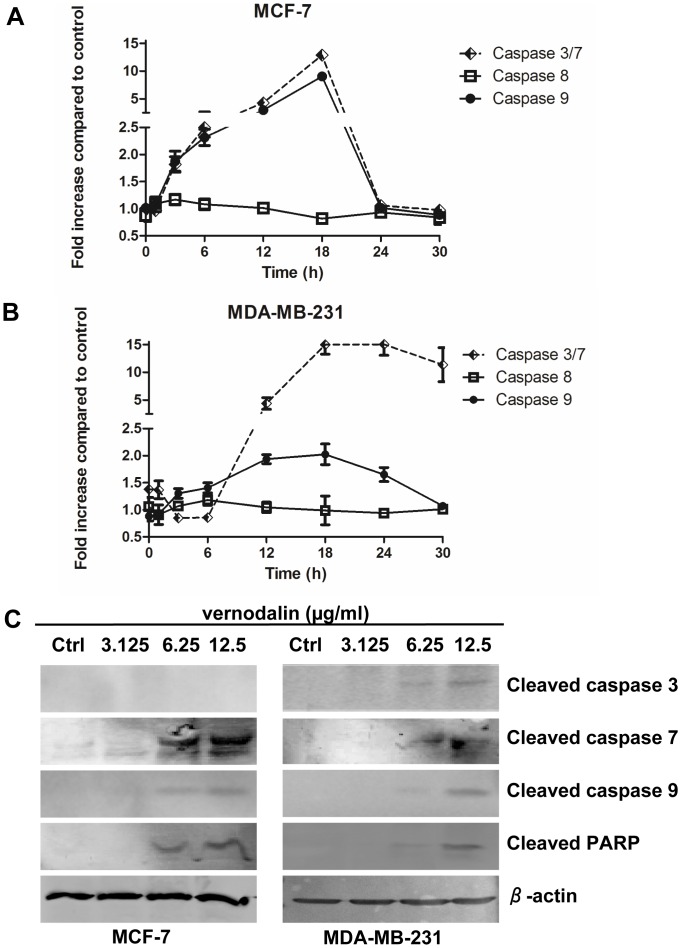
Vernodalin induces apoptosis through intrinsic caspase pathway. (A, B) Caspase-3/7, -8 and -9 activities in the vernodalin (6.25 µg/ml)-treated (A) MCF-7 or (B) MDA-MB-231 cells were determined as fold increase in luminescence against vehicle (DMSO)-treated cells at various time intervals. Initial activation of caspase-9 was followed by gradual increment activity of caspase-3/7 after vernodalin treatment. Data were mean ± SD. (C) Western blot showing the expression levels of cleaved caspase-3, -7, -9 and cleaved PARP in MCF-7 or MDA-MB-231 cells treated with DMSO (control) or various concentration (3.125, 6.25 and 12.5 µg/ml) of vernodalin. β-actin served as a loading control. Data were representative of at least two similar experiments.

Apoptosis is a complex activity that mobilizes a number of molecules and is classified into caspase-dependent or caspase-independent mechanisms. Caspase-dependent pathway can be further divided into extrinsic or intrinsic pathway, as determined by involvement of caspase-8 or caspase-9, respectively. Both intrinsic and extrinsic pathway involved activation of caspase-3/7 which is important for inducing downstream DNA cleavage molecules. To examine the molecular mechanism underlying apoptosis process, we stained cells with aminoluciferin-labeled substrate of caspase and determined the caspase-3/7, -8, -9 activities by measuring the luminescence intensities every three hours. As shown in Figure 13A, we observed a gradual increased of caspase-9 and caspase-3/7 activity, which peaked at 18 hours in both MCF-7 and MDA-MB-231 cells treated with 6.25 µg/ml of vernodalin (Figure 13A and 13B). The activity of caspase-3/7 increased significantly from 6 to 12 hours, but remained high even after 30 hours of treatment, indicating a more latent effect of vernodalin in MDA-MB-231 cells (Figure 13B). In contrast, there were no significant changes in the activity of caspase-8 for the time span of 30 hours in vernodalin treated-MCF-7 or MDA-MB-231 cells. Our data suggested that vernodalin induced activation of intrinsic caspase pathway in both breast cancer cell lines.

To examine whether caspases and the downstream PARP molecule were involved in vernodalin-induced apoptosis, we performed Western blot analysis using cell lysates of untreated/vernodalin-treated MCF-7 or MDA-MB-231 cells. Results indicated that vernodalin dose-dependently caused cleavage of caspase-7 and -9 in MCF-7, whereas caspase-3, -7 and -9 were activated in MDA-MB-231 cells (Figure 13C). On the other hand, PARP cleavage was also detected in both cells, suggesting involvement of caspase cascade and PARP inactivation in vernodalin-mediated apoptosis (Figure 13C).

### Vernodalin Downregulates Anti-apoptotic Molecules

**Figure 14:**
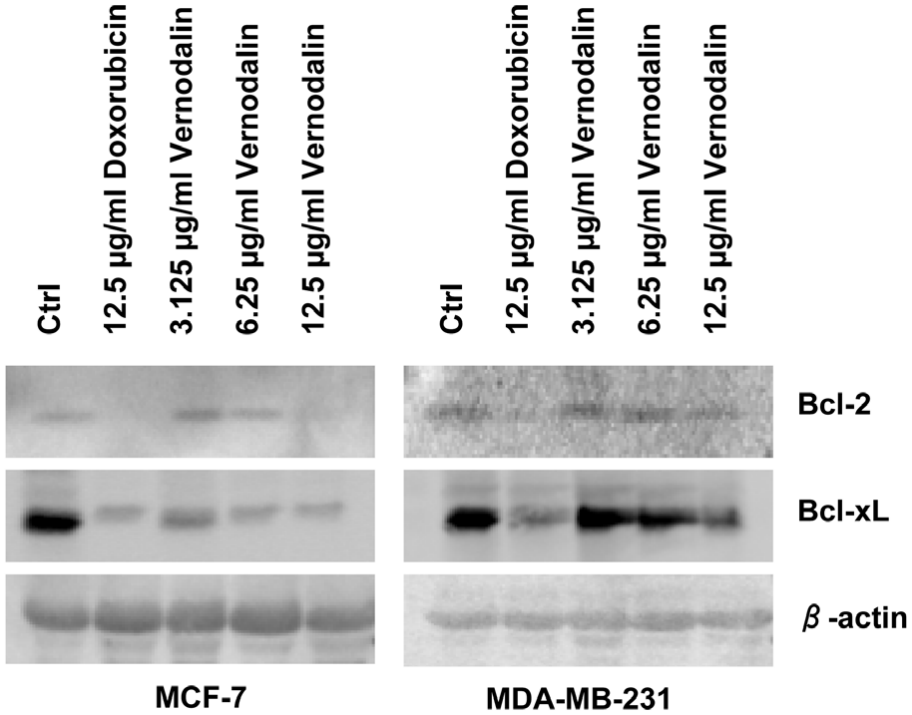
Vernodalin reduces expression of pro-survival molecules. MCF-7 and MDA-MB-231 cells were treated with control DMSO, standard drug doxorubicin (12.5 µg/ml) or various concentrations of vernodalin (3.125, 6.25, 12.5 µg/ml). Western blot showing the expression levels of the pro-survival molecules Bcl-2 and Bcl-xL in untreated and treated breast cancer cells. β-actin served as a loading control. Decreased Bcl-2 and Bcl-xL protein levels were observed upon doxorubicin or vernodalin treatment. Data were representative of at least two similar experiments.

Cell survival is maintained by pro-survival (anti-apoptotic) molecules such as Bcl-2 and Bcl-xL. To examine if the vernodalin initiated apoptosis by affecting the cellular level of these molecules, we performed Western blot analysis using control or vernodalin-treated breast cancer cells. Cells were also treated with a standard drug doxorubicin as a positive control of apoptosis induction. Our data showed that vernodalin dose-dependently reduced the expression level of Bcl-2 and Bcl-xL (Figure 14).

## Discussion

In this study, chloroform extract of the seed of *C. anthelmintica* (CACF) was tested for its cytotoxicity effect on MCF-7 human breast cancer cells. We showed that CACF effectively inhibited cell growth of MCF-7. CACF-treated cells exhibited morphological hallmarks of apoptosis including cell shrinkage, lost of cytoskeletal F-actin structure, higher stain with apoptotic marker annexin V and DNA damage. In fact, defects in apoptotic pathway are thought to contribute to a number of human malignancies [27]. Thus, anti-cancer agents that induce apoptosis is one of the efficient strategies in cancer chemotherapy [28].

Bioassay-guided isolation is a procedure whereby extract is chromatographically fractionated until a pure and active compound is isolated. Each fraction produce during the process is evaluated in bioassay system (e.g. *in vitro* cell culture, *in vivo* rat or zebrafish model etc) and the active fractions are chosen for further purification [29], [30], [31]. In this study, bioassay guided isolation of CACF extract led us to the identification of a potent compound vernodalin. Vernodalin is a sesquiterpene lactone isolated from various plant species including the seeds of *C. anthelmintica* [32]. Vernodalin exhibits anti-malarial and anti-bacterial activities and is a constituent of *Vernonia amygdalina* (Compositae), a plant ingested by wild chimpanzees sometimes suffering from parasite-related diseases in the Mahale Mountains National Park, Tanzania [33], [34], [35]. So far, only two studies have been done on anti-cancer effect of vernodalin, which demonstrated cytotoxic activity on melanoma and ovarian cancer cell lines [36] and human carcinoma of the nasopharynx (KB) [37]. These two studies mainly examined cell viability of vernodalin treated cancer cells by MTT based assays and less information was available on anti-cancer mechanism by vernodalin. To the best of our knowledge, this is the first report on cytotoxicity and mechanism of vernodalin on human breast cancer cells.

12,13-dihydroxyoleic acid (2), a compound isolated from CACF-C (a moderate active fraction) did not show inhibitory activity on breast cancer cells. The moderate activity of CACF-C could be due to the presence of trace quantity of vernodalin (Figure 5B). The structure of the compound was confirmed by comparing its spectral data (MS, ^1^H NMR, ^13^C NMR) with those reported for a synthetic 12,13-dihydroxyoleic acid [38]. The MS spectrum (Figure S2A) showed ions at *m/z* 182 and 131 corresponding to the allylic cleavage and indicated the double bond at C_9_ and C_10_ (Figure S2B). Fragmentation ions at *m/z* 213 and 157 corresponding to the alpha cleavage on the either side of the OH groups which confirmed their positions at C_12_ and C_13_ [38], [39]. A characteristic peak at m/z 85 (100%) as a result of ions formed through C_4_ and C_5_ cleavage and losing two protons form [C_4_H_7_O_2_^•^]. This is the first time for the compound (2) to be reported as secondary metabolite in plant. 12,13-dihydroxyoleic acid (2) is normally synthesized by acetolysis of vernolic acid, an epoxy fatty acid obtained from *C. anthelmintica* oil [40]. Hydroxy fatty acids are important in industry for the production of oleochemicals [38], [39].

We showed that vernodalin induced cell cycle arrest in breast cancer cells. Cell cycle progression is a hallmark for cell proliferation. Deregulation of cell cycle has been linked with cancer initiation and progression [41]. Thus, cell cycle has emerged as one of the attractive therapeutic target in the treatment of cancer. Nevertheless, siRNA or small molecule inhibitors that target cell cycle have been developed, for example flavopiridol is the first cell cycle inhibitor to be tested in clinical trials [42], [43]. To date, most of the chemotherapeutic agents caused cell cycle arrest either at G0/G1 or the G2/M stage, whereas cell cycle arrest at the S-phase is rare. For instance, synthetically methoxylated analogue of resveratrol induces G1 cell cycle arrest of human breast carcinoma MCF-7 cells [44] whereas curcumin induced G2/M cell cycle arrest in cisplatin resistant ovarian cancer cells [45]. The cell cycle is controlled by a group of cyclin family proteins called cyclin-dependent kinase (CDKs) enzymes [46]. The regulation of CDKs activities is achieved by their association with cyclin partners and kinases, phosphatases and specific inhibitors [46]. Future works are needed to examine the detail mechanism of cell cycle arrest in vernodalin-treated breast cancer cells.

ROS are either free radicals or reactive anions containing oxygen atoms, such as oxygen ions and peroxides. ROS could be a by-product of aerobic respiration, tissue-specific enzyme or microsomal cytochrome P450 metabolism of xenobiotic compounds [47]. High level of ROS can destroy the integrity of plasma membrane, affects dynamic of actin cytoskeleton and causes DNA damage, cumulatively known as oxidative stress [48], [49], [50]. Interestingly, we observed that ROS production in vernodalin-treated human breast cancer cells were 2–4 fold higher compared to control. Although harmful to cells, the anti-cancer effect of several conventional treatments such as ionizing radiation, etoposide or arsenates rely on their ability to stimulate ROS production, which modulate cellular redox balance leading to oxidative stress, destabilization of mitochondria and subsequently induction of apoptosis [51]. For example, etoposide caused severe ROS accumulation preferentially in the human glioblastoma-astrocytoma cells and elevated ROS rendered these cells highly sensitive to cell death [52].

Studies have shown that mitochondria played a key role in the apoptotic process [53], [54], [55]. Changes in the MMP (Δψm) increase the release of apoptogenic factors such as cytochrome c from the outer mitochondria membrane space into the cytosol. Released cytochrome c then form apoptosome with pro-caspase-9, apoptotic protease activating factor-1 (Apaf-1) and ATP, which in turn activates downstream apoptotic signal such as caspase-3/7 [56], [57]. However, malignant tumor cells predominantly produce ATP through glycolysis rather than oxidation of pyruvate in mitochondria like most normal cells, a phenomena known as Warburg effect [53]. Hence, tumor mitochondria are less susceptible to mitochondria membrane permeabilization rendering them more resistant to mitochondrial pathway of apoptosis [53]. In this report, we showed that vernodalin induced attenuation of MMP (Δψm) possibly through ROS production, which promote mitochondria membrane permeabilization and subsequent induction of apoptosis.

Caspases is a family of cysteine proteases that is divided into executioner caspases such as caspase-3 or -7, and initiator caspases, such as caspase-8 and -9 [57]. Initiator caspase-8 is known to be activated through extrinsic pathway, whereas caspase-9 is activated in the event of mitochondrial cytochrome c leakage [57]. Both initiator caspases can activate caspase-3 or -7, which commit cells to apoptosis [58]. Incubation with vernodalin causes a time-dependent activation of caspase-9, while caspase-8 activities remained at basal level. The increase in caspase-9 activity was concomitant with the increase in caspase-3/7 activity. These results suggest that vernodalin induced apoptosis via mitochondrial-dependent intrinsic pathway. Of note, caspase-3/7 cleaves several target proteins, one of which is DNA repair enzyme, PARP. Interestingly, DNA fragmentation was detected in vernodalin-treated MCF-7 and MDA-MB-231 cells. Since MCF-7 is deficient in caspase-3 expression, it is possible that DNA fragmentation could be mediated by activation of caspase-7 and PARP cleavage, as shown previously by other studies [59], [60].

Members of the Bcl-2 family are major regulators of cell death or cell survival. Bcl-2 and Bcl-xL act as apoptosis inhibitors in the cells. Our data showed that vernodalin treatment reduced expression of pro-survival/anti-apoptotic proteins Bcl-2 and Bcl-xL, implying the relevance of Bcl-2 family proteins for breast cancer cell survival. Another study by Shimizu *et al.* highlighted the importance of Bc1-2 and Bcl-xL in protecting mitochondria against loss of function during apoptosis and some forms of necrotic cell death [61]. Presence of Bcl-2 in mitochondria blocks cell death by inhibiting apoptosis-associated release of cytochrome c from the mitochondria [62], or by regulating ion flux [63]. Whereas Bcl-xL interacts with Apaf1 to prevent apoptosis by inhibiting Apaf1 dependent activation of caspase 9 [64]. Therefore, downregulation of Bcl-2 and Bcl-xL upon vernodalin treatment could lead to loss of MMP which facilitated cytochrome c release and activation of caspase cascade.

Estrogen stimulates proliferation of various breast cancer cells via estrogen receptors (ER). Studies show that compounds such as phytoestrogens, alkylphenols, organochlorine pesticides and phthalates could bind to estrogen receptors and mediate estrogen responses [65], [66], whereas polycyclic aromatic hydrocarbons (PAHs) or dioxin binds to aryl hydrocarbon receptor (AhR) which forms complex with ER [67]. To investigate whether the anti-proliferative effect by vernodalin was ER-dependent, we performed TR-FRET assays to examine the binding ability of vernodalin to ER-α and ER-β. However, we did not find any significant reduction of TR-FRET signal by vernodalin even at the highest concentration, 200 µg/ml (data not shown). In fact, MTT or Alamar blue cell viability assays showed comparable IC_50_ values between the two breast cancer cell-lines, MCF-7 (ER positive) and MDA-MB-231 (ER negative) 24 h after treatment with vernodalin (Figure 7 and S1B). Based on these findings, we propose that the presence of ER has no significant effect on vernodalin-induced cell growth inhibitory activity.

In conclusion, this report showed that CACF has profound activity against MCF-7 human breast cancer cell line. Through bioassay guided isolation, we identified vernodalin as the active compound responsible for the anti-cancer property in CACF. Our collective data suggest that vernodalin inhibits cell growth of MCF-7 and MDA-MB-231 breast cancer cells through induction of cell cycle arrest and apoptosis. Vernodalin induces apoptosis by generating ROS and downregulating pro-survival molecules Bcl-2 and Bcl-xL. These processes subsequently lead to attenuation of MMP and cytochrome c release. Release of cytochrome c activates caspase cascade and PARP cleavage to execute apoptotic program through fragmentation of chromatin DNA. The findings in this report indicate potential therapeutic value of vernodalin and further research in animal tumor models is necessary to confirm its anti-cancer activity *in vivo*.

## Supporting Information

Figure S1 Proliferation assay by Alamar blue assay. (A) MCF-7 cells were treated with vehicle (DMSO) or various concentrations (0.195, 0.39, 0.78, 1.56, 3.125, 6.25, 12.5, 25, 50 µg/ml) of CACF for 24 hours. After treatment, Alamar blue stain was added into culture medium for 2 hours (10% of total volume). Cell viability was determined by Alamar blue staining assay (AbD Serotec, Oxford, UK). The fluorescent intensity was measured with Bio-Tek Synergy H4 hybrid microplate reader (Bio-Tek, US) at 590 nm emission (560 nm excitation). Cell viability was calculated according to manufacturer’s manual. IC_50_ value for CACF-treated MCF-7 was 7.6±0.5 µg/ml, IC_50_ value for doxorubicin-treated MCF-7 was 3.4±0.5 µg/ml. (B) MCF-7, MDA-MB-231 and primary mammary epithelial cells were treated with various concentrations of vernodalin for 24 hours. Cell viability was determined as described above. MCF-7 (IC_50_ = 2.1±0.8 µg/ml), MDA-MB-231 (IC_50_ = 3.8±0.4 µg/ml), and primary mammary epithelial cells (IC_50_ = 14.2±1.3 µg/ml).
(TIF)

Figure S2 Mass spectra of 12,13-dihydroxyoleic acid. (A) MS spectrum of 12,13-dihydroxyoleic acid **(2)**. (B) MS fragmentation pattern of 12,13-dihydroxyoleic acid **(2)**. The MS spectrum showed ions at *m/z* 182 and 131 corresponding to the allylic cleavage and indicated the double bond at C_9_ and C_10_.
(TIF)
