# Supplementary figures and images for: Induction of Apoptosis in Human Breast Cancer Cells via Caspase Pathway by Vernodalin Isolated from Centratherum anthelminticum (L.) Seeds
Source: PLoS One. 2013 Feb 20;8(2):e56643. doi: 10.1371/journal.pone.0056643 (PMC3577860; doi:10.1371/journal.pone.0056643)

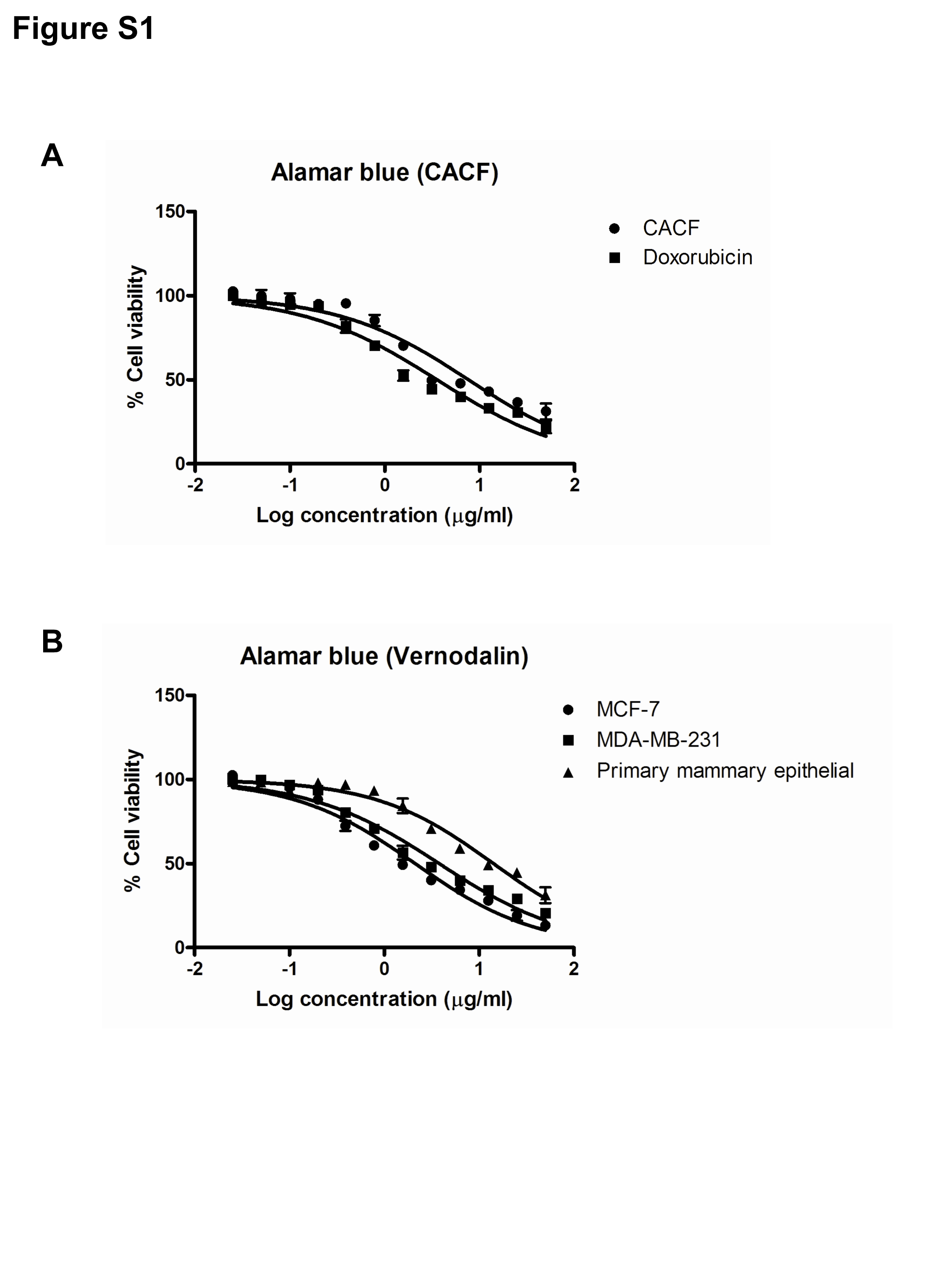

Supplement: Figure S1 — Proliferation assay by Alamar blue assay. (A) MCF-7 cells were treated with vehicle (DMSO) or various concentrations (0.195, 0.39, 0.78, 1.56, 3.125, 6.25, 12.5, 25, 50 µg/ml) of CACF for 24 hours. After treatment, Alamar blue stain was added into culture medium for 2 hours (10% of total volume). Cell viability was determined by Alamar blue staining assay (AbD Serotec, Oxford, UK). The fluorescent intensity was measured with Bio-Tek Synergy H4 hybrid microplate reader (Bio-Tek, US) at 590 nm emission (560 nm excitation). Cell viability was calculated according to manufacturer’s manual. IC50 value for CACF-treated MCF-7 was 7.6±0.5 µg/ml, IC50 value for doxorubicin-treated MCF-7 was 3.4±0.5 µg/ml. (B) MCF-7, MDA-MB-231 and primary mammary epithelial cells were treated with various concentrations of vernodalin for 24 hours. Cell viability was determined as described above. MCF-7 (IC50 = 2.1±0.8 µg/ml), MDA-MB-231 (IC50 = 3.8±0.4 µg/ml), and primary mammary epithelial cells (IC50 = 14.2±1.3 µg/ml). (TIF) [file pone.0056643.s001.tif]

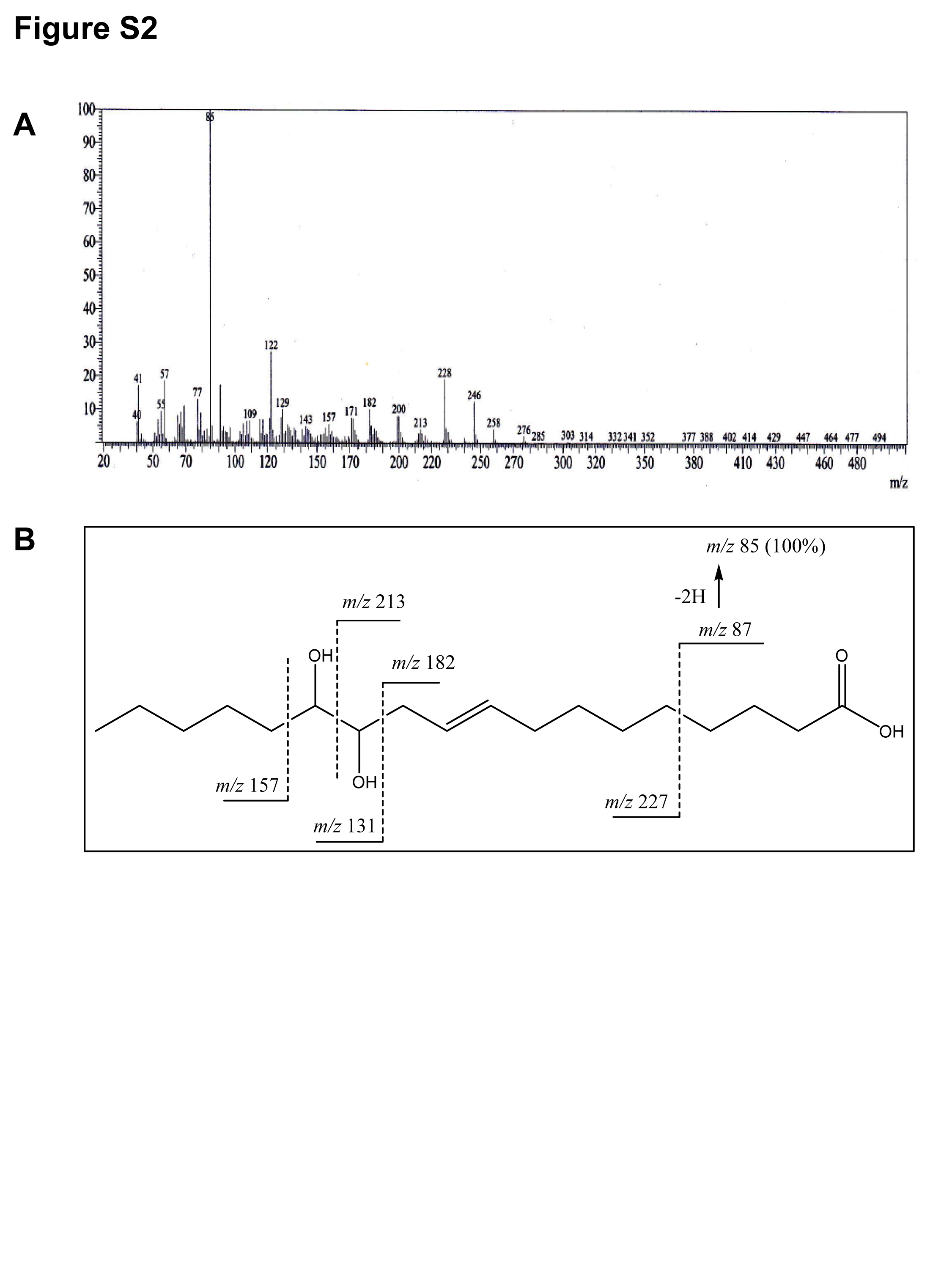

Supplement: Figure S2 — Mass spectra of 12,13-dihydroxyoleic acid. (A) MS spectrum of 12,13-dihydroxyoleic acid (2). (B) MS fragmentation pattern of 12,13-dihydroxyoleic acid (2). The MS spectrum showed ions at m/z 182 and 131 corresponding to the allylic cleavage and indicated the double bond at C9 and C10. (TIF) [file pone.0056643.s002.tif]
